# Dupilumab for atopic manifestations in pediatric patients with inborn errors of immunity: efficacy and safety in a genetically diverse cohort

**DOI:** 10.3389/fimmu.2025.1715919

**Published:** 2025-11-21

**Authors:** Najla Alsediq, Thekra Algholaiqa, Ghadah Alnami, Abdulrahman N. AlJaber

**Affiliations:** 1 Division of Pediatric Allergy and Immunology, Department of Pediatrics, King Abdullah Specialized Children’s Hospital, King Abdulaziz Medical City, Riyadh, Saudi Arabia; 2 King Abdullah International Medical Research Center, Ministry of National Guard-Health Affairs, Riyadh, Saudi Arabia; 3 College of Medicine, King Saud bin Abdulaziz University for Health Sciences, Riyadh, Saudi Arabia

**Keywords:** inborn errors of immunity, atopic dermatitis, dupilumab, asthma, atopy

## Abstract

**Background:**

Inborn Errors of Immunity (IEI) are monogenic disorders that predispose patients to infections, autoimmunity, atopy, and malignancies. Severe Th2-driven atopic dermatitis and asthma are common in several IEIs, including DOCK8 deficiency, STAT3 loss-of-function, and Wiskott–Aldrich syndrome, often refractory to conventional therapies. Dupilumab, a monoclonal antibody targeting IL-4Rα, has shown promise in managing recalcitrant atopic dermatitis and asthma, but data in immunocompromised pediatric populations remain limited.

**Methods:**

We conducted a single-center, retrospective observational cohort study with prospective follow-up of ten pediatric patients with genetically confirmed IEI and atopic manifestations. Patients received subcutaneous dupilumab (300 mg every 4 weeks for atopic dermatitis; every 2 weeks for severe asthma). Outcomes were assessed using the Eczema Area and Severity Index (EASI), Dermatology Life Quality Index (DLQI), Childhood Asthma Control Test (C-ACT), eosinophil counts, and infection frequency across four time points (baseline, 6 months, 12 months, >18 months).

**Results:**

The cohort included ten patients (6 males, 4 females) with mutations in DOCK8 (n=5), CARD11 (n=2), STAT3 LOF (n=1), ADA (n=1), and PEPD (n=1). Of ten patients, nine received dupilumab for atopic dermatitis. Baseline EASI and DLQI scores were 34.33 and 25.0, respectively. Following dupilumab therapy, mean EASI decreased to 2.67 (p<0.001) and DLQI improved to 2.67 (p<0.001), with sustained response observed over 18 months. Peripheral eosinophil counts declined from 2.0×10^9^/L to 0.45×10^9^/L (p<0.001). One patient with severe asthma achieved C-ACT improvement from 10 to 26 with no subsequent exacerbations. Treatment was well-tolerated, with only mild conjunctivitis or transient hypereosinophilia reported. Notably, skin infections resolved, and no systemic or opportunistic infections were observed.

**Conclusion:**

Dupilumab is a safe and highly effective therapeutic option for pediatric patients with IEI-associated atopic manifestations, improving clinical outcomes, quality of life, and infection profile while preserving immune function. These findings support broader application of dupilumab in patients with IEI and warrant further investigation in larger, multicenter studies.

## Introduction

Inborn Errors of Immunity (IEI) are genetic disorders characterized by increased susceptibility to infections, autoimmunity, autoinflammation, atopy, and malignancies. According to the latest classification by the International Union of Immunological Societies (IUIS), 559 monogenic defects have been linked to IEI (1). An atopic phenotype is common in certain disorders, such as DOCK8 deficiency, STAT3 hyper-immunoglobulin E syndrome, and Wiskott–Aldrich syndrome (1). Atopic manifestations typically include dermatitis, asthma, and food allergy. Notably, severe atopic dermatitis may be the first clinical sign of an underlying immunodeficiency. Atopic dermatitis associated with IEI can be caused by multiple mechanisms. Skewing toward Th2 pathway and enhancement of allergic inflammation is a common mechanism that can be observed in DOCK8 deficiency and CARD11 deficiency (2–6). Additionally, defects in Th17/IL-17 axis weaken the skin barrier function by decreasing antimicrobial peptide levels. This can explain the frequent colonization of *Staphylococcus aureus* and *Candida* in patients with atopy associated with IEI (7). Previous studies have shown that atopic dermatitis associated with IEI may be refractory to standard therapies, including emollients and topical corticosteroids (2–6). The underlying immunodeficiency limits the use of systemic therapies in these patients due to the risk of further immune suppression. Targeted biological therapy offers an effective and safer alternative for the treatment of atopic dermatitis. Dupilumab, a monoclonal antibody that targets the IL-4Rα subunit, inhibits signaling of both IL-4 and IL-13. Dupilumab was approved by the Food and Drug Administration (FDA) for the treatment of moderate-to-severe atopic dermatitis in children aged six months and older (8). Experience with dupilumab in immunocompromised patients exhibiting concomitant atopic manifestations remains primarily limited to case reports and small series. The use of dupilumab for the treatment of recalcitrant atopic dermatitis in patients with confirmed immunodeficiency was first reported in 2021 (5). In that report, two children with DOCK8 deficiency and dermatitis unresponsive to conventional therapies were started on dupilumab and showed significant improvement (5). Subsequently, the therapeutic benefits of dupilumab have been reported in controlling severe and extensive atopic dermatitis in patients with a range of primary immunodeficiencies, including STAT3 loss-of-function (LOF), autosomal recessive (AR) ZNF341 deficiency, DOCK8 mutations, autosomal dominant (AD) CARD11 LOF, X-linked agammaglobulinemia, Wiskott–Aldrich syndrome, ARPC1B deficiency, STAT6 gain-of-function (GOF), cytotoxic T lymphocyte antigen-4 insufficiency, and Netherton syndrome (2–6, 9–16). Dupilumab was generally well-tolerated and resulted in significant clinical improvement (6). Herein, we report the efficacy and safety profile of dupilumab in a cohort of ten pediatric patients with confirmed IEI and associated atopy. Our study—the first from the Middle East to evaluate dupilumab use in patients with IEI, to the best of our knowledge—demonstrates sustained efficacy and a favorable safety profile over long-term follow-up, including in two genetic defects for which dupilumab use has not been previously reported.

## Methods

### Study design and setting

A single-center, retrospective observational cohort study with prospective follow-up was conducted at the Pediatric Allergy and Immunology Division at King Abdullah Specialized Children’s Hospital in Riyadh, Saudi Arabia. Data were collected between January 2022 and August 2025. Participants were followed across four predefined time points: pre-treatment (baseline), post-1 (6 months), post-2 (12 months), and post-3 (>18 months). The study was approved by King Abdullah International Medical Research Center (IRB number: 0000070524).

### Study sample

All pediatric patients followed by the Pediatric Allergy and Immunology service with a molecular diagnosis of inborn errors of immunity and moderate-to-severe atopy refractory to conventional therapy, in whom dupilumab was initiated, were included. Atopic dermatitis was diagnosed based on Hanifin and Rajka criteria; asthma was diagnosed using Global Initiative for Asthma (GINA) guidelines, including spirometry when applicable; and food allergy was confirmed through clinical history, skin prick testing, specific IgE, and/or oral food challenge.

### Data collection

Data were retrieved from the institutional electronic medical record system. For each patient, demographic information, consanguinity status, family history, diagnosis, genetic mutation, skin manifestations, history of skin and respiratory infections, atopic symptoms, comorbidities, and concurrent treatments (e.g., intravenous immunoglobulin or prophylactic antibiotics) were recorded. In addition, serum IgE levels, absolute eosinophil counts, and specific disease scores were collected at four predefined time points.

### Intervention and outcomes

Patients received subcutaneous dupilumab according to body weight and clinical indication. Nine patients received 300 mg every 4 weeks for atopic dermatitis, while one patient with uncontrolled asthma received 300 mg every 2 weeks. The treatment regimen adhered to established pediatric guidelines and was consistent with protocols reported in previous studies. Dupilumab was administered in the hospital’s outpatient clinic under nursing supervision. Follow-up assessments were conducted after treatment initiation. Clinical photography and physician evaluations were employed to assess skin improvement. Patients were closely monitored for adverse reactions and infection-related complications.

The primary endpoints were improvement in skin disease severity, assessed using the Eczema Area and Severity Index (EASI), and quality of life, evaluated with the Dermatology Life Quality Index (DLQI). Asthma control was measured using the Childhood Asthma Control Test (C-ACT). Secondary endpoints included changes in absolute eosinophil counts, reduction in skin infection frequency, and assessment of adverse effects and overall tolerability of dupilumab.

### Statistical analysis

Continuous variables were summarized as mean ± standard deviation or median, as appropriate, while categorical variables were expressed as counts and percentages. Paired t-tests were used for normally distributed variables to compare pre- and post-treatment values, and Wilcoxon signed-rank tests were applied for non-normally distributed data. Repeated measures ANOVA was employed to assess overall differences across four time points for each subject. Tukey’s *post hoc* tests were performed to identify which specific time points differed significantly. A two-sided p-value <0.05 was considered statistically significant. All analyses were performed using SPSS version 28, with supplementary *post hoc* calculations and visualizations generated in Python.

## Results

This cohort includes a total of ten patients, six males and four females. Eight patients were born to consanguineous parents. The identified genetic mutations were diverse and included *DOCK8* (5 patients), *CARD11* (2 patients), *STAT3* LOF (1 patient), *ADA* (1 patient), and *PEPD* (1 patient). All patients had dermatitis associated with their underlying syndrome. Eight patients experienced recurrent documented skin infections. The distribution of infections was as follows: bacterial skin infections (impetigo and skin abscesses) occurred in all eight patients; viral skin infections—including herpes simplex virus (HSV), human papillomavirus (HPV), molluscum contagiosum, and varicella-zoster virus (VZV)—were observed in four patients; and candidiasis was documented in two patients. Additionally, six patients had recurrent respiratory infections and four had recurrent otitis media. Comorbidities included bronchiectasis (1 patient), rickets (1 patient), vitiligo (1 patient), and failure to thrive (2 patients). Elevated total IgE levels were detected in 9 of 10 cases, while peripheral eosinophilia was present in 8 of 10 cases.

Atopic manifestations were observed in all patients. Four patients were receiving immunoglobulin replacement therapy, and five were on prophylactic antibiotics. Demographic characteristics, family history, genetic findings, infection profile, and atopic features are summarized in **Table 1**.

**Table 1:** Demographic data, family history, mutation analysis, infection details, and atopies.

Patient no.	Age (y)	Gender	Consanguinity	Gene	Mutation	Mutation reference	Skin infections	Sinopulmonary infections	Systemic manifestations	Atopies	Immunoglobulin replacement/antibiotic prophylaxis	HSCT
1	6	Male	Yes	DOCK8	c.54-24125G>A, p.?	Not reported	–	Recurrent URTI	–	AD, FA, Drug Allergy, Asthma, AR	–	No
2	6	Male	Yes	DOCK8	c.949C>T, p.(Arg317*)	Reported (PMID: 19776401, 31980526)	Bacterial skin infections	Recurrent URTI, recurrent bacterial and viral pneumonia, recurrent OM	–	AD, Asthma, FA	IVIG Bactrim	No
3	22	Male	Yes	CARD11	c.1091G>A, p.(Arg364His)	Reported (PMID: PMC4167767)	–	–	Vitiligo	AD, FA	–	No
4	17	Female	Yes	CARD11	c.1091G>A, p.(Arg364His)	Reported (PMID: PMC4167767)	Bacterial skin infections	–	–	AD, FA	–	No
5	11	Male	Yes	DOCK8	c.(404 + 1_405-1)_(827 + 1_828-1)del	Not reported	Recurrent bacterial skin infections, viral skin infections, fungal skin infections	Recurrent URTI, recurrent OM	FTT	AD, FA	SCIG Bactrim	No
6	6	Female	Yes	DOCK8	c.(404 + 1_405-1)_(827 + 1_828-1)del	Not reported	Recurrent bacterial skin infections, viral skin infections, fungal skin infections	Recurrent URTI, recurrent OM	–	AD, FA	SCIG Bactrim	No
7	4	Male	Yes	ADA	c.247G>A, p.(Ala83Thr)	Reported (PMID: 7599635, 9758612)	Recurrent bacterial skin infections	–	Rickets	AD, FA, Drug Allergy	Bactrim	No
8	13	Female	Yes	DOCK8	c.894 + 2T>A	Not reported	Recurrent bacterial skin infections, viral skin infections	Recurrent URTI, recurrent OM	FTT	AD, FA, Drug Allergy	–	Yes
9	12	Male	No	PEPD	c.1354G>A, p.(Glu452Lys)	Reported (PMID: 25460580)	Recurrent bacterial skin infections, viral skin infections	–	–	AD, Asthma	–	No
10	16	Female	No	STAT3 LOF	Unavailable	Unavailable	Recurrent bacterial abscesses	Recurrent bacterial pneumonia	Bronchiectasis	AD, FA	IVIG Bactrim Itraconazole	No

HSCT, hematopoietic stem cell transplant; AD, atopic dermatitis; FA, food allergy; AR, allergic rhinitis; IVIG, intravenous immunoglobulin; SCIG, subcutaneous immunoglobulin; OM, otitis media; URTI, upper respiratory tract infection; FTT, Failure To Thrive.

**Figure 1:**
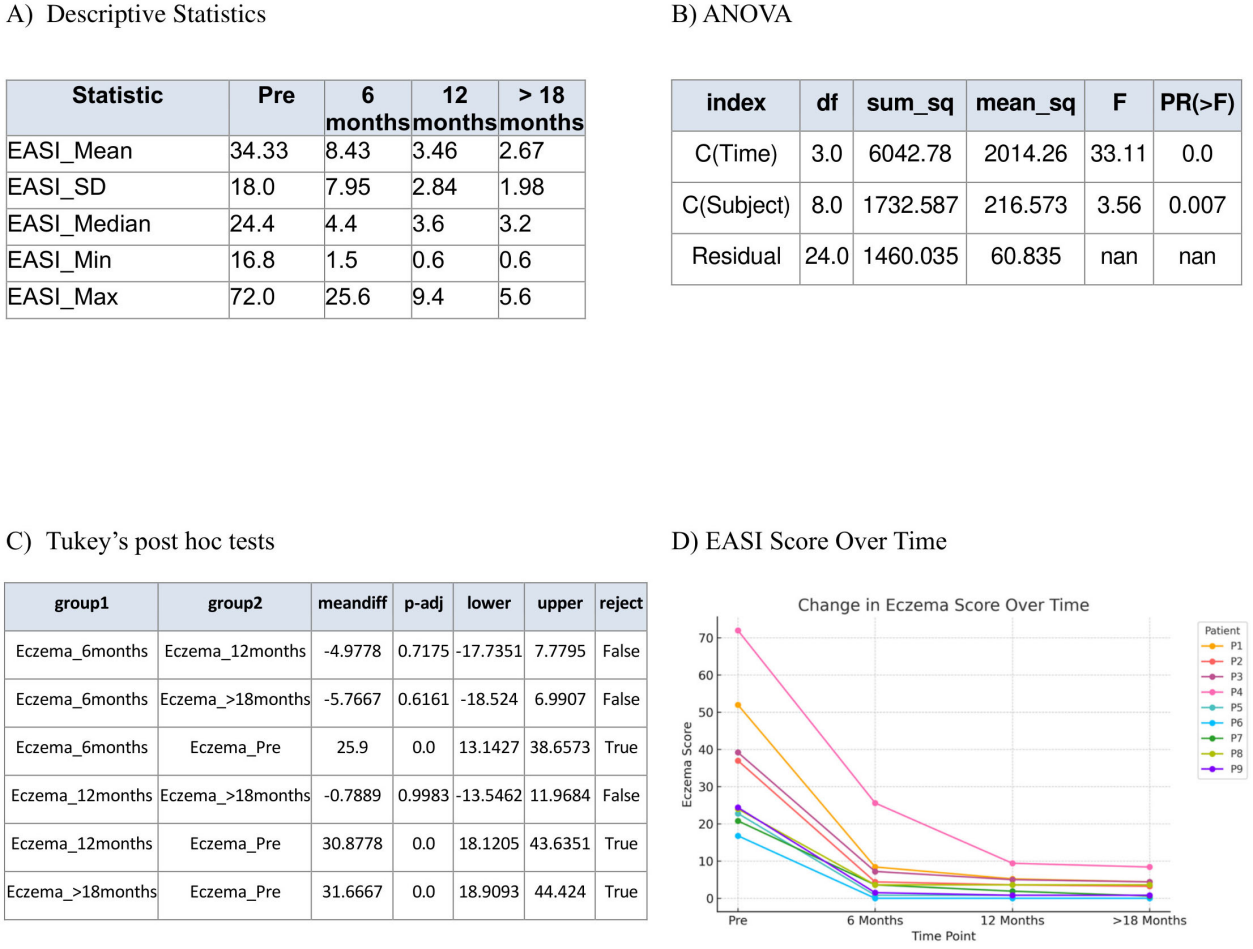
Eczema Area and Severity Index (EASI). **(A)** Descriptive statistics of EASI score in pre-treatment with dupilumab, 6 months post treatment, 12 months post treatment, and >18 months post treatment. **(B)** Repeated measures ANOVA. **(C)** Tukey’s *post hoc* tests. **(D)** Demonstration of the decrease in EASI score over time.

**Figure 2:**
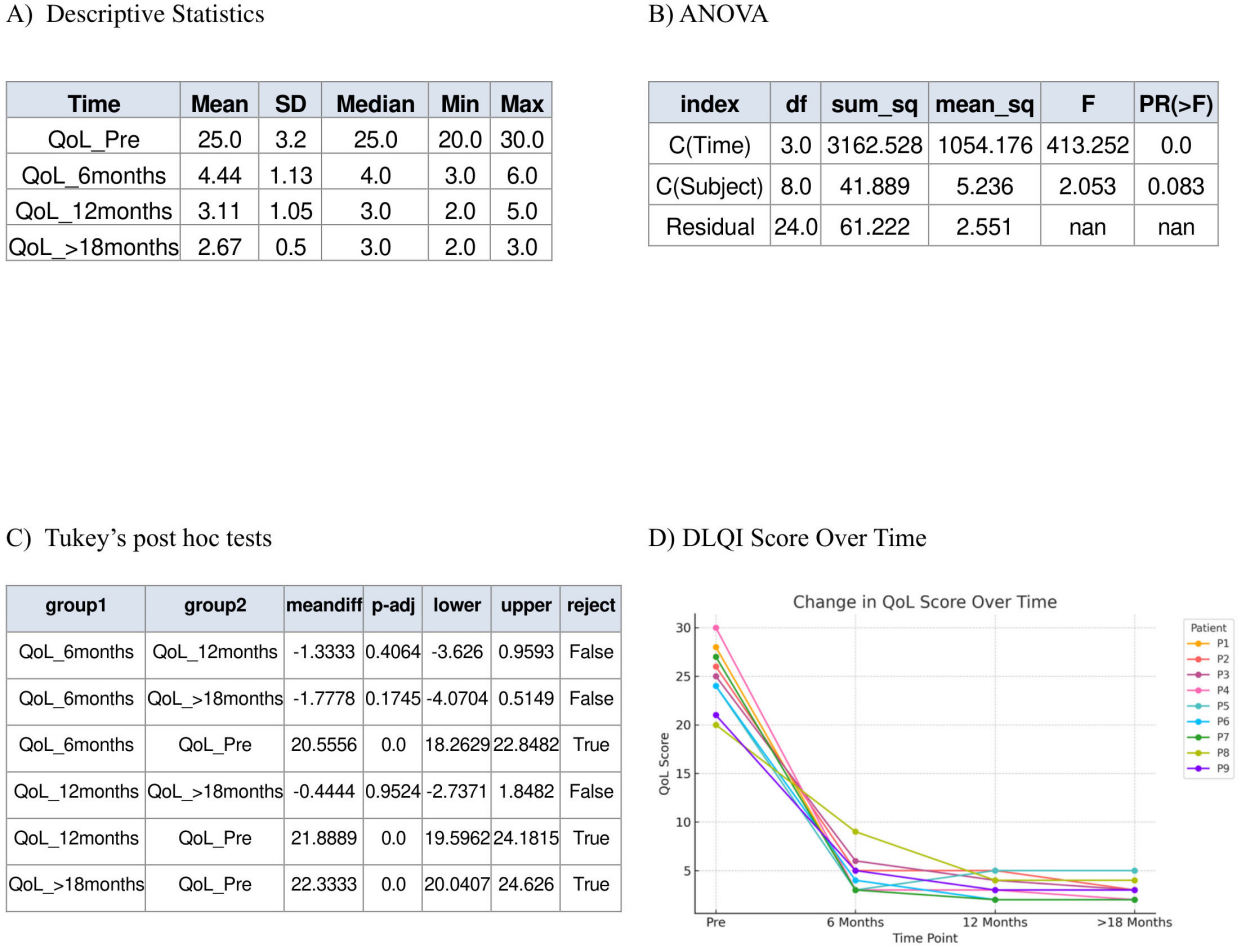
Dermatology Life Quality Index (DLQI). **(A)** Descriptive statistics of DLQI score in pre-treatment with dupilumab, 6 months post treatment, 12 months post treatment, and >18 months post treatment. **(B)** Repeated measures ANOVA. **(C)** Tukey’s *post hoc* tests. **(D)** Demonstration of the decrease in DLQI score over time.

**Figure 3:**
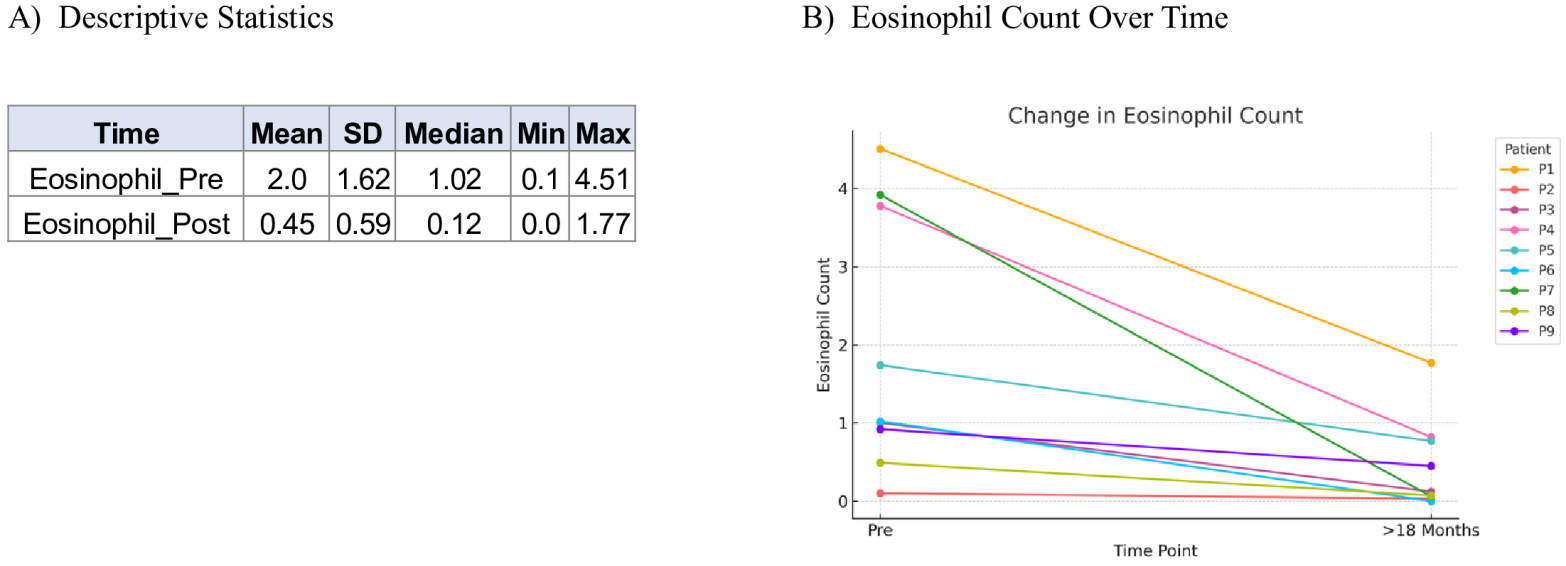
Eosinophil count. **(A)** Descriptive statistics of eosinophil count (10^9^/L) in pre-treatment with dupilumab and post-treatment phase. **(B)** Demonstration of the decrease in eosinophil counts between pre-treatment and >18 months post-treatment with dupilumab.

**Figure 4:**
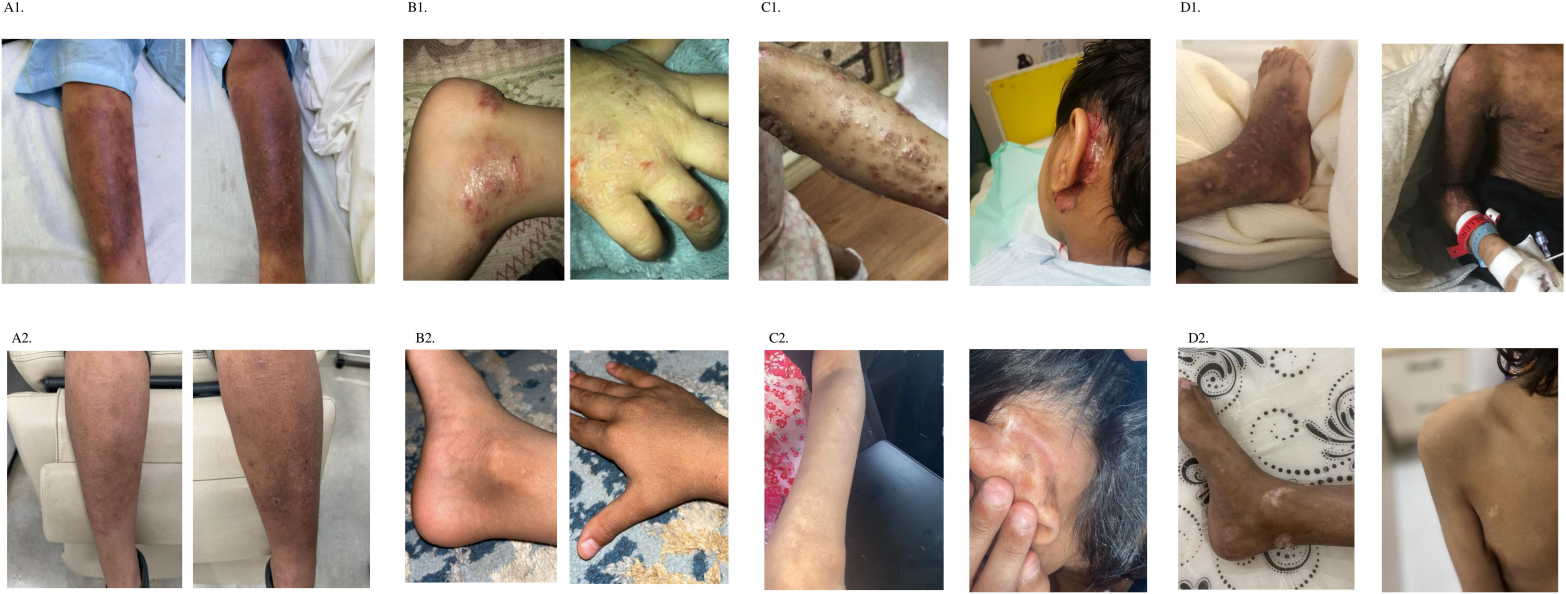
Representative photographs of patients’ skin manifestations. **(A)** Improvement in cutaneous manifestations from pre-treatment **(A1)** to post-treatment with dupilumab **(A2)** in PEPD patient. **(B)** Demonstration of the improvement in skin manifestations between pre-treatment **(B1)** and post-treatment with dupilumab **(B2)** in ADA patient. **(C)** Improvement in cutaneous manifestations from pre-treatment **(C1)** to post-treatment with dupilumab **(C2)** in DOCK8 patient. **(D)** Demonstration of the improvement in skin manifestations between pre-treatment **(D1)** and post-treatment with dupilumab **(D2)** in DOCK8 patient.

Nine patients presented with moderate-to-severe eczema refractory to topical therapy, with a baseline mean EASI score of 34.33. All patients initiated subcutaneous dupilumab at a dose of 300 mg every four weeks. Following treatment, the mean EASI score decreased to 2.67 (p < 0.001). Repeated measures ANOVA revealed a statistically significant effect of time (p < 0.001) demonstrating that treatment with dupilumab had a measurable effect over time. Tukey’s *post hoc* test showed that the greatest reduction in EASI scores occurred during the first six months of treatment (pre- vs. post-1), indicating a rapid therapeutic response followed by sustainability of effect (**Figure 1**). Mean DLQI score improved significantly, from 25.0 at baseline to 2.67 post-treatment (p < 0.001). Repeated measures ANOVA revealed a highly significant time effect (p < 0.001) confirming that patients’ perceived quality of life changed significantly throughout the treatment period. Tukey’s *post hoc* test showed that the largest and most statistically significant changes occurred between the pre-treatment phase and each post-treatment phase, indicating that patients experienced meaningful improvements in their daily life functioning early on, and these improvements were sustained (**Figure 2**). Similarly, the mean eosinophil counts declined from 2.0 × 10^9^/L at baseline to 0.45 × 10^9^/L after treatment (p < 0.001) reflecting the biological impact of dupilumab on inflammation (**Figure 3**). Representative photographs of patients’ skin manifestations are presented in **Figure 4**.

One patient presented with severe asthma refractory to inhaled corticosteroids and long-acting beta-agonists (Patient No. 2), with a baseline C-ACT score of 10. The patient experienced recurrent asthma exacerbations, necessitating multiple emergency room visits and pediatric intensive care unit (PICU) admissions. Treatment was initiated with subcutaneous dupilumab at a dose of 300 mg every two weeks. Following treatment, C-ACT score improved to 26, and the patient experienced no further asthma exacerbations requiring hospitalization.

Treatment was generally well-tolerated, with only mild adverse effects reported, including conjunctivitis in a single patient (Patient No. 1) and transient hypereosinophilia in two patients (Patients No. 5 and No. 8). The frequency of skin infections improved markedly following dupilumab therapy; none of the treated patients experienced further skin infections after initiating treatment. Importantly, no systemic or opportunistic infections were documented during therapy.

## Discussion

Atopy in the context of inborn errors of immunity provides a unique framework for elucidating immune dysregulation and the molecular pathways implicated in allergic diseases. As broader phenotypic patterns are increasingly recognized, atopy has emerged as a feature of numerous IEIs and, in certain cases, may represent the primary clinical manifestation (17). Atopic dermatitis in the context of IEIs often presents with early onset, a recurrent or relapsing course, and a suboptimal response to conventional therapies (6). Management of IEI-related dermatitis is often complicated by the presence of concurrent cutaneous viral infections and heightened susceptibility to bacterial infections, which may be exacerbated by the use of topical or systemic corticosteroids (5). In addition, patients with underlying IEI exhibit persistent activation of Th2-related cytokines, in contrast to atopic dermatitis, which typically follows a relapsing–remitting course and may be influenced by exogenous triggers (5). The underlying immune dysregulation in IEI—including Th2 polarization, impaired barrier repair, and altered microbial defense—may limit the effectiveness of topical corticosteroids and calcineurin inhibitors. This highlights why targeted cytokine blockade with dupilumab offers a more sustained and specific therapeutic benefit in this population (17).

This study provides real-world evidence supporting the safety and efficacy of dupilumab in pediatric patients with genetically confirmed IEI presenting with severe atopic dermatitis and associated allergic manifestations. Our results confirm and expand upon prior reports, while offering new insights into immunopathological mechanisms, clinical applicability, and therapeutic safety in this unique and vulnerable patient population.

Dupilumab has demonstrated sustained long-term safety in the management of atopic dermatitis, with accumulating evidence indicating a significant reduction in infection risk among treated patients (18). By inhibiting type 2 cytokine signaling, dupilumab markedly improves dermatitis manifestations and restores epidermal barrier function through increased expression of filaggrin and tight-junction proteins, leading to reduced transepidermal water loss and enhanced activity of antimicrobial peptides (3). Additionally, it suppresses epidermal hyperplasia and modulates immune activity by downregulating T-cell and dendritic cell function (4). Furthermore, dupilumab may enhance host defense against infections through downregulation of Th2-driven inflammation and facilitation of Th1 expansion. Consequently, increased infection susceptibility represents a potential therapeutic indication for dupilumab in the management of atopic dermatitis among immunocompromised individuals (2). A key advantage of dupilumab in this population is its ability to selectively target specific cytokines without inducing additional immunosuppression. Given its approval for use in children as young as six months, dupilumab offers a much-needed treatment option for managing skin-related complications in patients with primary immunodeficiencies.

The use of dupilumab in our cohort of patients with IEI revealed promising outcomes for those struggling with severe eczema and compromised skin barrier function. All patients in our study experienced substantial improvements in skin inflammation, quality of life, and a reduction in skin infections. All patients achieved at least EASI-75, and more than 50% achieved EASI-90. There was a notable improvement in DLQI scores. Our findings are consistent with emerging reports of dupilumab use in monogenic immunodeficiencies.

Our report also offers a longer follow-up period of dupilumab treatment compared with previously published cases in patients with IEI, with our cohort followed for more than 18 months of continuous therapy. Patients demonstrated a sustained clinical response, with no documented systemic or opportunistic infections during therapy. On the contrary, the observed resolution of recurrent skin infections in our patients further supports the role of dupilumab in enhancing the natural barrier function of the skin—a crucial benefit for this vulnerable group. The safety profile of dupilumab, which minimizes additional immunosuppression, is a significant advantage when treating immunocompromised patients.

One patient underwent successful hematopoietic stem cell transplantation (HSCT), resulting in complete remission after the transplantation and consequent discontinuation of dupilumab treatment with no recurrence of symptoms. Dupilumab may serve as a bridge therapy, alleviating inflammatory conditions and skin infections while patients await hematopoietic stem cell transplantation.

Our cohort also included two patients with ADA and PEPD deficiencies presenting with associated dermatitis. Both patients demonstrated significant clinical improvement without notable adverse effects, thereby expanding the spectrum of IEIs potentially amenable to dupilumab therapy, as its use in these specific disorders has not been previously reported.

Overall, in our cohort, dupilumab has demonstrated both efficacy and safety in the treatment of atopic dermatitis in patients with IEI, although it does not provide a definitive therapy for the underlying immunodeficiency. These findings underscore the transformative potential of dupilumab in treating patients with IEI and severe atopic manifestations.

To our knowledge, this is the first study from the Middle East evaluating the efficacy and safety of dupilumab for the treatment of IEI-associated atopic manifestations, highlighting its therapeutic potential in the region. Although promising, this study has several limitations, including the small sample size, single-center design, and relatively short duration of follow-up. Future investigations should address these limitations by establishing multicenter registries and conducting prospective trials that include larger, ethnically diverse cohorts. Long-term follow-up will be essential to determine the durability of efficacy, assess the impact on infection susceptibility, and evaluate safety. Efforts to identify predictive biomarkers—such as periostin, Thymus and activation-regulated chemokine (TARC), IL-13, and epithelial barrier proteins including filaggrin—are needed to guide personalized treatment (19–21). Integration of dupilumab into transplantation strategies, particularly as a bridge-to-HSCT therapy in DOCK8, WAS, and other transplant-eligible IEIs, also warrants exploration. In addition, microbiome and transcriptomic profiling may help to clarify how dupilumab modulates immune–epithelial interactions in genetically immunodeficient patients. Finally, health economics analysis will be critical for informing access policies in regions characterized by high consanguinity and a significant rare disease burden.

We conclude that dupilumab represents a highly effective and safe therapeutic option for managing IEI-related atopic manifestations. Dupilumab improves clinical outcomes while preserving immune function, offering a unique therapeutic advantage that marks a significant step forward in the management of this complex patient population. We anticipate that other forms of primary immunodeficiency—especially those characterized by elevated IgE levels and eosinophilia that share similar dermatologic manifestations—may similarly benefit from this therapy.

## Data Availability

The original contributions presented in the study are included in the article/Supplementary Material. Further inquiries can be directed to the corresponding author.

## References

[1] Bousfiha AA Jeddane L Moundir A Poli MC Aksentijevich I Cunningham-Rundles C . The 2024 update of IUIS phenotypic classification of human inborn errors of immunity. J Hum Immun. (2025) 1:e20250002. doi: 10.70962/jhi.20250002, PMID: 36198931

[2] Fan YH Lin TL Sun HL Pan HH Ku MS Lue KH . Successful treatment of atopic dermatitis with dupilumab in the setting of X-linked agammaglobulinemia. J Allergy Clin Immunol Pract. (2022) 10:3032–4. doi: 10.1016/j.jaip.2022.07.026, PMID: 35961615

[3] Wang HJ Yang TT Lan CE . Dupilumab treatment of eczema in a child with STAT3 hyper-immunoglobulin E syndrome. J Eur Acad Dermatol Venereol. (2022) 36:e367–9. doi: 10.1111/jdv.17889, PMID: 34927771

[4] Alzahrani F Miller HK Sacco K Dupuy E . Severe eczema in Wiskott-Aldrich syndrome-related disorder successfully treated with dupilumab. Pediatr Dermatol. (2024) 41:143–4. doi: 10.1111/pde.15397, PMID: 37469225

[5] Ollech A Mashiah J Lev A Simon AJ Somech R Adam E . Treatment options for DOCK8 deficiency-related severe dermatitis. J Dermatol. (2021) 48:1386–93. doi: 10.1111/1346-8138.15955, PMID: 34043252

[6] Zangari P Giancotta C Pacillo L Colantoni N Leone F Amodio D . Use of dupilumab for atopic dermatitis in pediatric and young adult patients with inborn errors of immunity. Pediatr Allergy Immunol. (2024) 35:e14215. doi: 10.1111/pai.14215, PMID: 39105363

[7] Hazime R Eddehbi FE El Mojadili S Lakhouaja N Souli I Salami A . Inborn errors of immunity and related microbiome. Front Immunol. (2022) :982772. doi: 10.3389/fimmu.2022.982772, PMID: 36177048 PMC9513548

[8] Food and Drug Administration (FDA) . FDA website(2022). Available online at: https://www.accessdata.fda.gov/drugsatfda_docs/label/2022/761055s042lbl.pdf?utm_medium=email&utm_source=transaction (Accessed August 8, 2025).

[9] Staudacher O Krüger R Kölsch U Thee S Gratopp A Wahn V . Relieving job: Dupilumab in autosomal dominant STAT3 hyper-IgE syndrome. J Allergy Clin Immunol Pract. (2022) 10:349–351.e1. doi: 10.1016/j.jaip.2021.08.042, PMID: 34536614

[10] Gualdi G Lougaris V Amerio P Petruzzellis A Parodi A Burlando M . Prurigo-like atopic dermatitis in a child with CARD11-associated severe combined immunodeficiency successfully treated with dupilumab. Pediatr Dermatol. (2024) 41:158–9. doi: 10.1111/pde.15453, PMID: 37888582

[11] Lévy R Béziat V Barbieux C Puel A Bourrat E Casanova JL . Efficacy of dupilumab for controlling severe atopic dermatitis in a patient with hyper-IgE syndrome. J Clin Immunol. (2020) 40:418–20. doi: 10.1007/s10875-020-00751-4, PMID: 31993867 PMC9642001

[12] Arruda LK Cordeiro DL Langer SS Koenigham-Santos M Calado RT Dias MM . Efficacy of dupilumab for the treatment of severe skin disease in cytotoxic T lymphocyte antigen-4 insufficiency: A role of type 2 inflammation? J Allergy Clin Immunol Glob. (2022) 2:114–7. doi: 10.1016/j.jacig.2022.08.004, PMID: 37780100 PMC10509893

[13] Fujishima C Munemoto S Hioki C Sasaki H Yoshida H Yamamoto T . Successful dupilumab therapy for atopic dermatitis in a patient with X-linked agammaglobulinaemia. Eur J Dermatol. (2022) 32:416–7. doi: 10.1684/ejd.2022.4288, PMID: 36065552 PMC9660116

[14] Su CJ Tseng HC . Treatment efficacy of dupilumab in a hyper-immunoglobulin E syndrome patient with severe atopic dermatitis. JAAD Case Rep. (2021) 11:60–2. doi: 10.1016/j.jdcr.2021.03.007, PMID: 33898684 PMC8054095

[15] Vollmuth Y Alelq NA Sattler F Schmidt S Hauck F . Dupilumab in a 9-week-old with netherton syndrome leads to deep symptom control. J Clin Immunol. (2024) 45:42. doi: 10.1007/s10875-024-01837-z, PMID: 39546052 PMC11568019

[16] Trombello S Jarisch A Willasch A Rettinger E Fekadu-Siebald J Holzinger D . Case report: Advanced age at transplantation and pre-emptive treatment with dupilumab in DOCK8 deficiency. Front Immunol. (2025) 15:1507494. doi: 10.3389/fimmu.2024.1507494, PMID: 39936153 PMC11810938

[17] Sams L Wijetilleka S Ponsford M Gennery A Jolles S . Atopic manifestations of inborn errors of immunity. Curr Opin Allergy Clin Immunol. (2023) 23:478–90. doi: 10.1097/ACI.0000000000000943, PMID: 37755421 PMC10621644

[18] Guttman-Yassky E Bissonnette R Ungar B Suárez-Fariñas M Ardeleanu M Esaki H . Dupilumab progressively improves systemic and cutaneous abnormalities in patients with atopic dermatitis. J Allergy Clin Immunol. (2019) 143:155–72. doi: 10.1016/j.jaci.2018.08.022, PMID: 30194992

[19] Maintz L Welchowski T Herrmann N Brauer J Traidl-Hoffmann C Havenith R . CK-CARE study group. IL-13, periostin and dipeptidyl-peptidase-4 reveal endotype-phenotype associations in atopic dermatitis. Allergy. (2023). doi: 10.1111/all.15647. Epub ahead of print, PMID: 36647778

[20] Halling AS Rinnov MR Ruge IF Gerner T Ravn NH Knudgaard MH . Skin TARC/CCL17 increase precedes the development of childhood atopic dermatitis. J Allergy Clin Immunol. (2023) 151:1550–7. doi: 10.1016/j.jaci.2022.11.023, PMID: 36572354

[21] Bakker D de Bruin-Weller M Drylewicz J van Wijk F Thijs J . Biomarkers in atopic dermatitis. J Allergy Clin Immunol. (2023) 151:1163–8. doi: 10.1016/j.jaci.2023.01.019, PMID: 36792449

